# Case Report: Accelerated parathyroid adenoma progression and hypercalcemia-driven neurotoxicity following pembrolizumab-based neoadjuvant therapy in triple-negative breast cancer: a case of multisystem immune-related adverse events

**DOI:** 10.3389/fonc.2025.1607957

**Published:** 2025-11-12

**Authors:** Yi Zeng, Yu Sun, Pengfei Qian

**Affiliations:** Department of Breast Surgery, The Affiliated Huizhou Hospital, Guangzhou Medical University, Huizhou, Guangdong, China

**Keywords:** breast cancer, pembrolizumab, parathyroid adenoma, hypercalcemia, immune-related adverse

## Abstract

This case report describes a 45-year-old TNBC patient (cT3N1M0 IIIA) with preexisting parathyroid adenoma who developed multisystem immune-related toxicities after neoadjuvant pembrolizumab chemotherapy. After two cycles, severe hepatotoxicity (ALT 1,090 U/L), grade 2 dermatitis, hypercalcemic crisis (iPTH 61.99 pmol/L), and meningeal thickening with intracranial hypertension emerged. Imaging revealed 134% parathyroid adenoma enlargement and diffuse meningeal enhancement. Emergency parathyroidectomy and corticosteroids normalized calcium and reversed neurological symptoms. Pathological complete response (pCR) occurred despite ICI discontinuation. This first-reported case suggests that PD-1 inhibitors may activate parathyroid microenvironments to drive adenoma growth. At the same time, calcium–ICI synergy could impair blood–brain barrier integrity, advocating calcium/neurological monitoring in ICI-treated endocrine disorder patients.

## Introduction

Triple-negative breast cancer (TNBC), characterized by the absence of actionable therapeutic targets, historically demonstrates suboptimal outcomes with conventional chemotherapy. Recent advances in PD-1/PD-L1 inhibitors combined with neoadjuvant chemotherapy have significantly improved pathological complete response (pCR) rates, emerging as the standard neoadjuvant approach for locally advanced TNBC (1). However, immune checkpoint inhibitor (ICI)-related multiorgan toxicities (hepatotoxicity, dermatitis, endocrine dysfunction) necessitate heightened vigilance, and their pathogenesis and individual susceptibility remain incompletely elucidated (2). This report details a locally advanced TNBC patient developing sequential grade 4 hepatotoxicity (ALT 1090 U/L), CTCAE grade 2 dermatitis, iPTH-driven hypercalcemia, and immune-mediated meningeal thickening following pembrolizumab chemotherapy. Complete multisystem toxicity resolution occurred postsurgical parathyroidectomy and ICI discontinuation, representing the first documentation of ICI-associated parathyroid adenoma acceleration and calcium-neurotoxicity synergy.

## Case description

**Figure 1:**
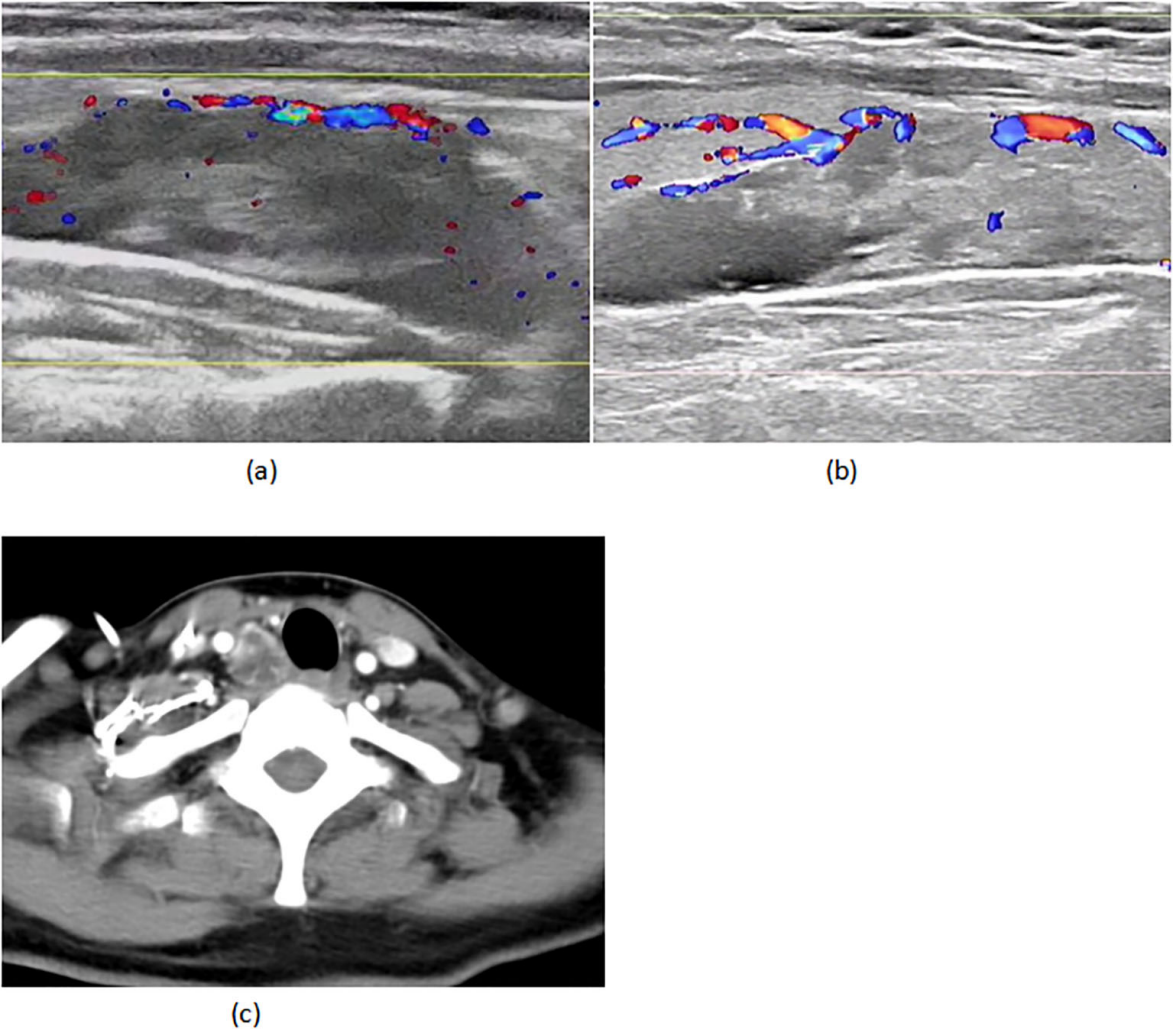
**(A)** 02–29 parathyroid ultrasound **(B)** 05-18 parathyroid ultrasound **(C)** 05–18 CT of the neck.

A 45-year-old woman with triple-negative breast cancer (cT3N1M0, stage IIIA) initiated neoadjuvant therapy (nab-paclitaxel/carboplatin/pembrolizumab) in March 2024. She achieved clinical partial response (cPR) after two well-tolerated cycles. Baseline evaluation revealed a right parathyroid mass (38×13mm, negative biopsy) (**Figure 1A**) with mild hypercalcemia (2.90 mmol/L; normal 2.10-2.60) and elevated iPTH (15.46 pmol/L; normal 1.6-6.9), suggesting parathyroid dysfunction.

**Figure 2:**
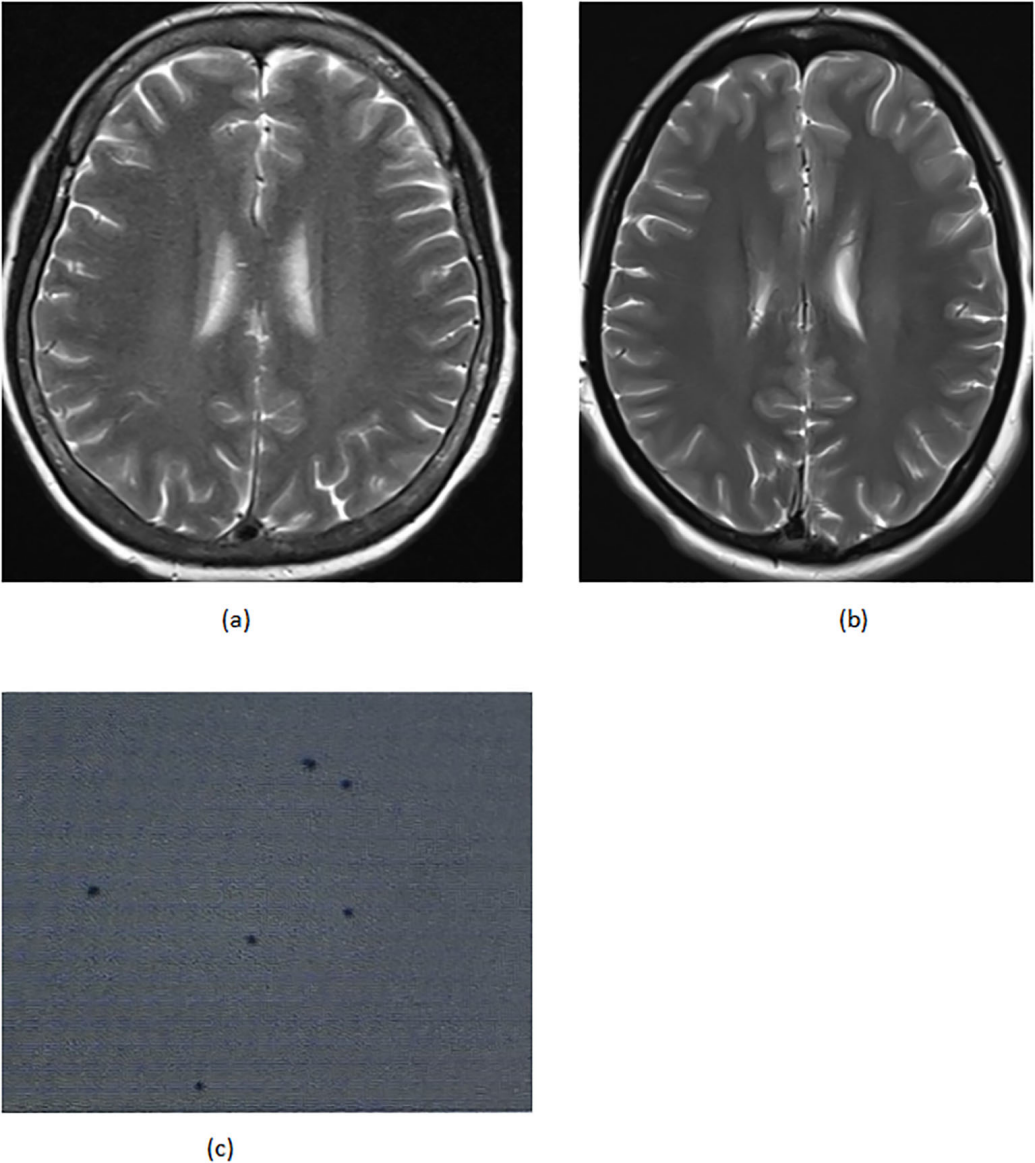
**(A)** 05–07 the brain MRI **(B)** 06–03 the brain MRI **(C)** 05–14 cerebrospinal fluid biopsy.

On April 22 (cycle 3 initiation), she developed grade 4 drug-induced liver injury (ALT 1090.3 U/L, AST 500.3 U/L). After excluding viral/autoimmune hepatitis, magnesium isoglycyrrhizinate therapy achieved partial hepatic recovery by May 1 (ALT 242.0 U/L, AST 81.5 U/L). Clinical status abruptly deteriorated on May 5 with confluent erythematous papules (CTCAE grade 2 rash), fever (39.0 °C), and leukopenia (WBC 2.1×10^9^/L). Corticosteroids and antihistamines gradually resolved cutaneous manifestations. On May 7, she presented with acute-onset severe headache, emesis, and hypertension (170/98 mmHg). Contrast-enhanced MRI demonstrated diffuse leptomeningeal enhancement along bilateral frontotemporal regions and tentorium cerebelli (**Figure 2A**). Cerebrospinal fluid analysis showed no malignant cells but elevated opening pressure (70 cmH_2_O) (**Figure 2C**), suggesting immune-related meningeal inflammation. Concurrent metabolic derangements progressed rapidly: hypercalcemia (3.22 mmol/L), iPTH surge (44.85 pmol/L, 290% baseline increase), and rising ALP (222 U/L) indicated hypercalcemic crisis. Emergency endocrinological intervention (salmon calcitonin/zoledronic acid/fluid resuscitation) transiently reduced calcium levels. The parathyroid mass enlarged to 51×25mm (134% volume increase) (**Figures 1B, C**) by late May, with iPTH rising to 61.99 pmol/L. Parathyroid scintigraphy confirmed functional adenoma. Right, parathyroidectomy on May 22 confirmed parathyroid adenoma histologically, with postoperative normalization of calcium (2.28 mmol/L) and iPTH. Given the temporal association of multisystem toxicity (hepatic, neurological, metabolic) with immunotherapy, pembrolizumab was discontinued in May while continuing dual chemotherapy for four cycles. Postoperative brain MRI on June 03 revealed complete resolution of meningeal thickening. (**Figure 2B**) Five-month follow-up showed no residual hepatic, dermatological, metabolic, or neurological sequelae. Left mastectomy on November 17 confirmed pathological complete response (pCR). The detailed treatment process is illustrated in **Table 1**.

**Table 1:** Treatment timeline chart.

Time	Key events and management
March	**Diagnosis**: cT3N1M0 IIIA TNBC; baseline parathyroid adenoma (38×13 mm, iPTH 15.46 pmol/L). **Treatment**: Nab-paclitaxel + carboplatin + pembrolizumab initiated.
April	**Cycle 2**: Partial response (cPR). **Pre-cycle 3 (Apr 22)**: Grade IV hepatotoxicity (ALT 1090 U/L). Action: Pembrolizumab paused; liver support.
May 1	**Liver recovery**: ALT 242 U/L. Continued hepatoprotective therapy.
May 5	**Immune toxicity**: Grade 2 rash, fever, leukopenia. **Action**: Glucocorticoids + antihistamines.
May 7	**Crisis**: - Meningitis (MRI: meningeal thickening). - Hypercalcemia (Ca²^+^; 3.22 mmol/L, iPTH 44.85 pmol/L). **Action**: ICP management, calcitonin + zoledronic acid.
May 22	**Surgery**: Parathyroid adenoma resected (volume ↑134%, iPTH 61.99 pmol/L). Post-op: Ca²^+^;/iPTH normalized.
May–Nov	**Adjusted therapy**: Pembrolizumab stopped; continued chemo (4 cycles). **Follow-up**: Brain MRI normal (June 03).
5-month follow-up	**Outcome**: No residual toxicity
Nov 17	**Mastectomy**: Pathologic complete response (pCR).

Bold value indicates key events.

## Discussion

This study presents the first documented case of accelerated parathyroid adenoma progression and immune-mediated leptomeningeal thickening associated with PD-1 inhibitor pembrolizumab during neoadjuvant therapy for triple-negative breast cancer (TNBC). The evolving clinical manifestations offer critical insights into immune checkpoint inhibitor (ICI) toxicity mechanisms. While immune-mediated hepatitis (IMH) and cutaneous reactions are well-established complications, ICI-induced central nervous system (CNS) toxicity such as meningeal inflammation and metabolic disturbances including hypercalcemia remain exceptionally rare in clinical practice.

In breast cancer management, hypercalcemia typically correlates with bone metastases or paraneoplastic syndromes (3, 4). Nevertheless, several case reports have suggested that pembrolizumab could trigger immune-related endocrine disorders (5), which may stimulate the production of PTHrP and calcitriol, and potentially cause hypocortisolemia. All these alterations can disturb calcium homeostasis, leading to the development of hypercalcemia. This suggests that pembrolizumab-associated hypercalcemia likely involves indirect mechanisms, rather than directly promoting hyperparathyroidism or cancer progression (6). Additionally, existing research indicates that the evidence linking pembrolizumab to parathyroid abnormalities is limited. There are only sporadic reports of patients developing hypoparathyroidism after treatment with pembrolizumab (7), leading to subsequent hypocalcemia (8). These observations align with findings reported in the KEYNOTE-189 and CHECKMATE-067 clinical trials (9). However, pembrolizumab presents a novel therapeutic avenue for patients with advanced parathyroid carcinoma exhibiting MSI-H or high TMB, demonstrating a significant reduction in tumor load following treatment. Nonetheless, the mechanism of action, which involves reversing immunosuppression, may unintentionally result in multiorgan dysfunction, encompassing hypercalcemic crisis and central neurotoxicity (10–12). In this case, the significant elevation of iPTH, the doubling of the adenoma volume, and the reversal of postoperative indicators strongly suggest a direct correlation between hyperparathyroidism and ICI exposure. Although no studies to date have indicated a direct relationship between PD-L1 and the growth of parathyroid adenomas, based on immunological mechanisms, we hypothesize that PD-1 inhibitors may block the PD-1/PD-L1 signaling pathway, thereby alleviating the immunosuppressive state within the tumor microenvironment, leading to abnormal proliferation of primary parathyroid adenoma cells and subsequent hyperparathyroidism (13). Concurrent systemic inflammation from ICI therapy could further stimulate parathyroid hormone hypersecretion (14, 15). These findings significantly expand the recognized spectrum of ICI-related endocrine toxicities, emphasizing the necessity for rigorous iPTH surveillance in patients with preexisting parathyroid abnormalities. Notably, the baseline assessment ruled out established factors for hyperparathyroidism in this patient, including chronic kidney disease (16), prior exposure to PTH-like agents (17), and denosumab administration (18), which strengthens the evidence for an association between pembrolizumab and the development of hyperparathyroidism. Nevertheless, since CDC73 genetic screening was not conducted for this patient, the possibility of CDC73-associated hyperparathyroidism cannot be excluded (19).

The neurological presentation—contrast-enhancing leptomeningeal thickening on MRI, intracranial hypertension (70 cmH_2_O), and acellular cerebrospinal fluid—aligns with classic ICI-associated aseptic meningitis (20, 21). Mechanistically, PD-1 inhibition may activate peripheral T cells capable of crossing the blood–brain barrier, subsequently releasing IFN-γ and other pro-inflammatory cytokines within meningeal tissues (22). Notably, coexisting hypercalcemia might synergistically increase vascular permeability, potentially exacerbating blood–brain barrier disruption and cerebral edema formation. This pathophysiological interplay underscores the importance of prioritizing immune-mediated toxicity over metastatic disease in I-CI-treated patients presenting with neurological symptoms, thereby avoiding diagnostic delays or inappropriate steroid administration.

The sequential emergence of grade 4 hepatotoxicity, CTCAE grade 2 dermatitis, hypercalcemic crisis, and meningeal inflammation reveals a potential temporal progression pattern of multiorgan ICI toxicity. Study limitations include the absence of PD-L1 expression analysis in resected adenoma tissue and detailed T-cell infiltration profiling. Future investigations employing multiomics approaches are required to elucidate molecular pathways linking ICI therapy to parathyroid dysregulation. Clinically, these findings mandate heightened vigilance for atypical ICI toxicities in patients with endocrine comorbidities, particularly emphasizing calcium/iPTH monitoring in populations predisposed to parathyroid dysfunction. The case further highlights the necessity for early multidisciplinary intervention when managing concurrent immune-mediated complications.

This paradigm-shifting case redefines the current understanding of ICI endocrine toxicity while illuminating the complex interplay between metabolic derangements and neuroinflammation in immunotherapy-related adverse events. According to the updated ASCO guidelines, aseptic meningitis is a rare neurological irAE, with typical symptoms including headache, photophobia, neck stiffness, and nausea. Diagnosis requires ruling out infectious causes via lumbar puncture. Management strategies include suspending ICPi, obtaining neurological consultation, and initiating corticosteroid therapy with a slow taper to prevent recurrence, which is consistent with the present case. While hyperparathyroidism is a relatively uncommon irAE, its occurrence might signal a more extensive endocrine-immune imbalance. This underscores the importance of evaluation by an endocrinology specialist to assess calcium and phosphate homeostasis and determine the potential need for hormonal intervention. In summary, the early detection of these irAEs, a multidisciplinary team approach (engaging medical oncology, neurology, and endocrinology), and adherence to guideline-recommended management are paramount for weighing the antitumor benefits against the potential toxicities, thereby optimizing patient prognosis. Further research is warranted to elucidate the pathogenesis of these uncommon irAEs and to investigate personalized therapeutic approaches (23).It serves as a critical reminder that expanding clinical awareness of rare ICI toxicities remains essential for optimizing cancer immunotherapy safety profiles.

While our investigation provides compelling evidence for a causal link between pembrolizumab exposure and the accelerated progression of parathyroid adenoma, based on meticulous clinical time-series data and the normalization of postoperative indicators, it is imperative to recognize that the intrinsic molecular mechanisms are not yet fully understood. The observed phenomenon of adenoma proliferation in this case is particularly remarkable, considering that PD-1 inhibitors are more commonly reported to induce hypoparathyroidism (24, 25). It remains uncertain whether this is a fortuitous association or attributable to distinct molecular features within the patient’s parathyroid adenoma (such as specific gene mutations or the immune microenvironment), which may have led to an anomalous reaction to checkpoint inhibitor therapy. The absence of PD-L1 expression assessment and comprehensive genomic profiling of the adenoma tissue means these questions are still unresolved. Future research efforts should concentrate on performing multiomics analyses on analogous cases to decipher the underlying pathogenic mechanisms, which is pivotal for comprehending these rare immune-related adverse events.

## Data Availability

The original contributions presented in the study are included in the article/**Supplementary Material**. Further inquiries can be directed to the corresponding author.

## References

[1] Mittendorf EA Zhang H Barrios CH Saji S Jung KH Hegg R . Neoadjuvant atezolizumab in combination with sequential nab-paclitaxel and anthracycline-based chemotherapy versus placebo and chemotherapy in patients with early-stage triple-negative breast cancer (IMpassion031): a randomised, double-blind, phase 3 trial. Lancet. (2020) 396:1090–100. doi: 10.1016/S0140-6736(20)31953-X, PMID: 32966830

[2] Lei C Kong X Li Y Yang H Zhang K Wang Z . PD-1/PD-L1 inhibitor - related adverse events and their management in breast cancer. J Cancer. (2024) 15:2770–87. doi: 10.7150/jca.85433, PMID: 38577606 PMC10988294

[3] Giannetta E Sesti F Modica R Grossrubatscher EM Guarnotta V Ragni A . Case report:Unmasking hypercalcemia in patients with neuroendocrine neoplasms. Experience from six Italian referral centers. Front Endocrinol. (2021) 12:665698. doi: 10.3389/fendo.2021.665698, PMID: 34093441 PMC8170398

[4] Guise TA Wysolmerski JJ . Cancer-associated hypercalcemia. N Engl J Med. (2022) 386:1443–51. doi: 10.1056/NEJMcp2113128, PMID: 35417639

[5] Lazzaroni M Angelini F Guglielmi R Novizio R Iadevaia A Andreadi A . Severe hypercalcemia following pembrolizumab therapy: A case report and A literature review. Endocr Metab Immune Disord Drug Targets. doi: 10.2174/0118715303409910250725043347, PMID: 40755097 PMC13284645

[6] Plessers S Mebis J De Moor N Wessels T Luyten D Requilé A . Hypercalcemia as an immune-related adverse event in a patient receiving nivolumab and ipilimumab for metastatic melanoma: A case report. Case Rep Oncol Med. (2025) 2025:8600200. doi: 10.1155/crom/8600200, PMID: 40689241 PMC12271711

[7] Lupi I Brancatella A Cetani F Latrofa F Kemp EH Marcocci C . Activating antibodies to the calcium-sensing receptor in immunotherapy-induced hypoparathyroidism. J Clin Endocrinol Metab. (2020) 105:dgaa092. doi: 10.1210/clinem/dgaa092, PMID: 32112105

[8] Trinh B Sanchez GO Herzig P Läubli H . Inflammation-induced hypoparathyroidism triggered by combination immune checkpoint blockade for melanoma. J Immunother Cancer. (2019) 7:52. doi: 10.1186/s40425-019-0528-x, PMID: 30791949 PMC6385398

[9] Nalluru SS Piranavan P Ning Y Ackula H Siddiqui AD Trivedi N . Hypocalcemia with immune checkpoint inhibitors: the disparity among various reports. Int J Endocrinol. (2020) 2020:7459268. doi: 10.1155/2020/7459268, PMID: 32587615 PMC7294349

[10] Park D Airi R Sherman M . Microsatellite instability driven metastatic parathyroid carcinoma managed with the anti-PD1 immunotherapy, pembrolizumab. BMJ Case Rep. (2020) 13:e235293. doi: 10.1136/bcr-2020-235293, PMID: 32967944 PMC7513558

[11] Teleanu MV Fuss CT Paramasivam N Pirmann S Mock A Terkamp C . Targeted therapy of advanced parathyroid carcinoma guided by genomic and transcriptomic profiling. Mol Oncol. (2023) 17:1343–55. doi: 10.1002/1878-0261.13398, PMID: 36808802 PMC10323885

[12] Katoh H Mitsuma T Okamoto R Naito K Tokito T Kikuchi M . Pembrolizumab with external radiation therapy effectively controlled TMB-high unresectable recurrent parathyroid cancer: a case report with review of literature. Endocr J. (2024) 71:1069–75. doi: 10.1507/endocrj.EJ24-0126, PMID: 38987211 PMC11778383

[13] Postow MA Sidlow R Hellmann MD . Immune-related adverse events associated with immune checkpoint blockade. N Engl J Med. (2018) 378:158–68. doi: 10.1056/NEJMra1703481, PMID: 29320654

[14] Tanaka T Narazaki M Kishimoto T . Immunothera⁃ peutic implications of IL-6 blockade for cytokine storm. Immunotherapy. (2016) 8:959–70. doi: 10.2217/imt-2016-0020, PMID: 27381687

[15] Tanaka R Okiyama N Okune M Ishitsuka Y Watanabe R Furuta J . Serum level of interleukin-6 is increased in nivolumab-associated psoriasiform dermatitis and tumor necrosis factor-α is a biomarker of nivolumab recativity. J Dermatol Sci. (2017) 86:71–3. doi: 10.1016/j.jdermsci.2016.12.019, PMID: 28069323

[16] Oxlund CS Hansen H Hansen S Rohold A . Progressive valvular calcifications with critical aortic stenosis in a 25-year-old woman with end-stage renal disease on haemodialysis: a case report. Eur Heart J Case Rep. (2021) 5:ytab061. doi: 10.1093/ehjcr/ytab061, PMID: 34345761 PMC8323062

[17] Solomon A Birkenfeld S . Rapid progression of aortic stenosis after initiation of teriparatide treatment: a case report. Cardiovasc Endocrinol Metab. (2020) 10:56–8. doi: 10.1097/XCE.0000000000000220, PMID: 33634257 PMC7901823

[18] Camponovo C Aubry-Rozier B Lamy O Gonzalez Rodriguez E . Hypercalcemia upon denosumab withdrawal in primary hyperparathyroidism: a case report and literature review. Osteoporos Int. (2020) 31:2485–91. doi: 10.1007/s00198-020-05676-7, PMID: 33057735 PMC7661408

[19] van der Tuin K Tops CMJ Adank MA Cobben JM Hamdy NAT Jongmans MC . CDC73-related disorders: clinical manifestations and case detection in primary hyperparathyroidism. J Clin Endocrinol Metab. (2017) 102:4534–40. doi: 10.1210/jc.2017-01249, PMID: 29040582

[20] Chang E Sabichi AL Sada YH . myasthenia gravis after nivolumab therapy for squamous cell carcinoma of the bladder. J Immunother. (2017) 40:114–6. doi: 10.1097/CJI.0000000000000161, PMID: 28234667

[21] Touat M Talmasov D Ricard D Psimaras D . Neurological toxicities associated with immune-checkpoint inhibi⁃ tors. Curr Opin Neurol. (2017) 30:659–68. doi: 10.1097/WCO.0000000000000503, PMID: 28938341

[22] Gu Y Menzies AM Long GV Fernando SL Herkes G . Immune mediated neuropathy following checkpoint immunotherapy. J Clin Neurosci. (2017) 45:14–7. doi: 10.1016/j.jocn.2017.07.014, PMID: 28765062

[23] Schneider BJ Naidoo J Santomasso BD Lacchetti C Adkins S Anadkat M . Management of immune-related adverse events in patients treated with immune checkpoint inhibitor therapy: ASCO guideline update. J Clin Oncol. (2021) 39:4073–126. doi: 10.1200/JCO.21.01440, PMID: 34724392

[24] Dadu R Rodgers TE Trinh VA Kemp EH Cubb TD Patel S . Calcium-sensing receptor autoantibody-mediated hypoparathyroidism associated with immune checkpoint inhibitor therapy: diagnosis and long-term follow-up. J Immunother Cancer. (2020) 8:e000687. doi: 10.1136/jitc-2020-000687, PMID: 32581059 PMC7319718

[25] Win MA Thein KZ Qdaisat A Yeung SJ . Acute symptomatic hypocalcemia from immune checkpoint therapy-induced hypoparathyroidism. Am J Emerg Med. (2017) 35:1039.e5–1039.e7. doi: 10.1016/j.ajem.2017.02.048, PMID: 28363614

